# Supplementary material for: Phenology and predictors of spring emergence for the Timber Rattlesnake (Crotalus horridus)
Source: PeerJ. 2023 Sep 25;11:e16044. doi: 10.7717/peerj.16044 (PMC10538278; doi:10.7717/peerj.16044)
Supplement: Supplemental Information 1 — We created 32 Generalized Logistic Mixed Effects Models (GLMMs) to examine the environmental drivers of daily surface presence of C. horridus at refugia in Jo Daviess (northern Illinois; n = 3) and Jersey (west-central Illinois; n = 3) counties during late winter and spring of 2018–2020. We chose the best model from the set for further analysis using Akaike’s Information Criterion corrected for small sample sizes (AICc). Descriptions of predictor variables are found in Table 1. [file peerj-11-16044-s001.docx]

| **Model** | **Parameters** |
| --- | --- |
| 1 | Intercept only |
| 2 | Global |
| 3 | Latitude + ADD^2^+ Min. Temp^2^ |
| 4 | Latitude * Min. Temp^2 +^ ADD^2^ |
| 5 | Latitude * ADD^2^ + Min. Temp^2^ |
| 6 | Latitude * Min. Temp^2^ |
| 7 | Latitude + Min. Temp^2^ |
| 8 | Latitude + ADD^2^ + Max. Temp^2^ |
| 9 | Latitude * Max. Temp^2^ + ADD^2^ |
| 10 | Latitude * ADD^2^ + Max. Temp^2^ |
| 11 | Latitude * Max. Temp^2^ |
| 12 | Latitude + Max. Temp^2^ |
| 13 | Latitude + ADD^2^ + Mean Temp^2^ |
| 14 | Latitude * Mean. Temp^2^ + ADD^2^ |
| 15 | Latitude * ADD^2^ + Mean Temp^2^ |
| 16 | Latitude * Mean Temp^2^ |
| 17 | Latitude + Mean Temp^2^ |
| 18 | Latitude + ADD^2^ + Min_5_. Temp^2^ |
| 19 | Latitude * Min_5_. Temp^2^ + ADD^2^ |
| 20 | Latitude * ADD^2^ + Min_5_. Temp^2^ |
| 21 | Latitude * Min_5_. Temp^2^ |
| 22 | Latitude + Min_5_. Temp^2^ |
| 23 | Latitude + ADD^2^ + Max_5_. Temp^2^ |
| 24 | Latitude * Max_5_. Temp^2^ + ADD^2^ |
| 25 | Latitude * ADD^2^ + Max_5_. Temp^2^ |
| 26 | Latitude * Max_5_. Temp^2^ |
| 27 | Latitude + Max_5_. Temp^2^ |
| 28 | Latitude * Day of Year^2^ |
| 29 | Latitude * Day of Year^2^ + Max. Temp^2^ |
| 30 | Latitude * Day of Year^2^ + Min. Temp^2^ |
| 31 | Latitude * Day of Year^2^ + Min_5_. Temp^2^ |
| 32 | Latitude * ADD^2^ |
