# Supplementary material for: Phenology and predictors of spring emergence for the Timber Rattlesnake (Crotalus horridus)
Source: PeerJ. 2023 Sep 25;11:e16044. doi: 10.7717/peerj.16044 (PMC10538278; doi:10.7717/peerj.16044)
Supplement: Supplemental Information 2 — We determined day of year (day 1 = 1-Jan) and corresponding date thresholds (TH) for each latitude (in 1° increments) using 14-day moving averages of predicted probabilities averaged across all years, allowing examination of general phenological trends (Fig. 6). We do not report probability threshold values >60% as 14 day moving averages did not typically exceed this value, and do not report probability threshold values of 0% as such occurrences were rare (i.e., there is always some probability of surface presence). Selection of probability thresholds should be determined by the appropriate authorities depending on the specific application (see “Conservation Implications” in the discussion) and should be treated as hypotheses until verified with empirical data. [file peerj-11-16044-s002.docx]

| **TH** | **Latitude (°)** | | | | | |
| --- | --- | --- | --- | --- | --- | --- |
|  | **37** | **38** | **39** | **40** | **41** | **42** |
| 5% | 54 (23-Feb) | 59 (28-Feb) | 69 (10-Mar) | 87 (28-Mar) | 90 (31-Mar) | 95 (5-Apr) |
| 10% | 56 (25-Feb) | 62 (3-Mar) | 84 (25-Mar) | 93 (3-Apr) | 94 (4-Apr) | 110 (20-Apr) |
| 15% | 65 (6-Mar) | 83 (24-Mar) | 85 (26-Mar) | 94 (4-Apr) | 95 (5-Apr) | 112 (22-Apr) |
| 20% | 80 (21-Mar) | 84 (25-Mar) | 87 (28-Mar) | 95 (5-Apr) | 96 (6-Apr) | 114 (24-Apr) |
| 25% | 82 (23-Mar) | 85 (26-Mar) | 90 (31-Mar) | 96 (6-Apr) | 96 (6-Apr) | 119 (29-Apr) |
| 30% | 85 (26-Mar) | 86 (27-Mar) | 94 (4-Apr) | 97 (7-Apr) | 99 (9-Apr) | 120 (30-Apr) |
| 35% | 86 (27-Mar) | 88 (29-Mar) | 95 (5-Apr) | 98 (8-Apr) | 111 (21-Apr) | 121 (1-May) |
| 40% | 90 (31-Mar) | 90 (31-Mar) | 95 (5-Apr) | 99 (9-Apr) | 112 (22-Apr) | 122 (2-May) |
| 45% | 91 (1-Apr) | 95 (5-Apr) | 96 (6-Apr) | 100 (10-Apr) | 113 (23-Apr) | 122 (2-May) |
| 50% | 92 (2-Apr) | 95 (5-Apr) | 98 (8-Apr) | 111 (21-Apr) | 116 (26-Apr) | 124 (4-May) |
| 55% | 95 (5-Apr) | 96 (6-Apr) | 98 (8-Apr) | 112 (22-Apr) | 119 (29-Apr) | 134 (14-May) |
| 60% | 96 (6-Apr) | 99 (9-Apr) | 99 (9-Apr) | 114 (24-Apr) | 121 (1-May) | 135 (15-May) |
